# CirComPara: A Multi-Method Comparative Bioinformatics Pipeline to Detect and Study circRNAs from RNA-seq Data

**DOI:** 10.3390/ncrna3010008

**Published:** 2017-02-10

**Authors:** Enrico Gaffo, Annagiulia Bonizzato, Geertruy te Kronnie, Stefania Bortoluzzi

**Affiliations:** 1 Department of Molecular Medicine, University of Padova, Padova 35131, Italy; enrico.gaffo@gmail.com; 2 Department of Women’s and Children’s Health, University of Padova, Padova 35128, Italy; annagiulia.bonizzato@gmail.com (A.B.); truustekronnie@gmail.com (G.t.K.)

**Keywords:** circular RNA, bioinformatics pipeline, RNA-seq, Quaking, monocytes, CirComPara

## Abstract

Circular RNAs (circRNAs) are generated by back-splicing of immature RNA forming covalently closed loops of intron/exon RNA molecules. Pervasiveness, evolutionary conservation, massive and regulated expression, and post-transcriptional regulatory roles of circRNAs in eukaryotes have been appreciated and described only recently. Moreover, being easily detectable disease markers, circRNAs undoubtedly represent a molecular class with high bearing on molecular pathobiology. CircRNAs can be detected from RNA-seq data using appropriate computational methods to identify the sequence reads spanning back-splice junctions that do not co-linearly map to the reference genome. To this end, several programs were developed and critical assessment of various strategies and tools suggested the combination of at least two methods as good practice to guarantee robust circRNA detection. Here, we present CirComPara (http://github.com/egaffo/CirComPara), an automated bioinformatics pipeline, to detect, quantify and annotate circRNAs from RNA-seq data using in parallel four different methods for back-splice identification. CirComPara also provides quantification of linear RNAs and gene expression, ultimately comparing and correlating circRNA and gene/transcript expression levels. We applied our method to RNA-seq data of monocyte and macrophage samples in relation to haploinsufficiency of the RNA-binding splicing factor Quaking (QKI). The biological relevance of the results, in terms of number, types and variations of circRNAs expressed, illustrates CirComPara potential to enlarge the knowledge of the transcriptome, adding details on the circRNAome, and facilitating further computational and experimental studies.

## 1. Introduction

Circular RNAs (circRNAs) are generated from immature RNA by a process called back-splicing where the 3′ and 5′ ends of linear RNA molecules are covalently joined in a non-collinear way forming RNA-loops. It has been estimated that circRNAs are produced from more than 10% of genes [1]. Circularity confers specific properties to circRNAs: they have longer half-lives compared with linear RNAs [2], tend to accumulate in cells with a low proliferation rate [3], and are resistant to RNase R. RNA circularization can engage single exons, two or more exons [4], both exon and intron sequences, or intronic sequences only [5]. A given gene can generate several circular isoforms and these isoforms may show distinct expression profiles [6]. Generation of circRNAs happens at the expense of their corresponding linear RNA isoforms and a correlation between exon skipping and circularization [7] has been demonstrated. Back-splicing adds complexity to alternative RNA splicing, and circRNA biogenesis and splicing are clearly interleaved processes. Thus, mutations or deregulation of splicing factors and/or alterations of *cis*-regulatory elements may impact circRNA biogenesis. Several splicing factors have been linked to circRNA expression: the RNA-editing enzyme *ADAR1* (adenosine deaminase RNA specific) antagonizes back-splicing, whereas muscleblind like splicing regulator (MBL) and the RNA-binding protein Quaking (QKI) both seem to promote circRNA levels [8]. As also suggested by their evolutionary conservation, a critical position of circRNAs in core biological processes starts to unfold. Like linear isoforms, circRNAs can act as competing endogenous RNAs that decoy miRNAs and indirectly regulate miRNA target gene and protein expression. Notably, circRNAs with multiple miRNA-binding sites are efficient miRNA sponges that participate in the regulation of specific cellular pathways [9,10,11,12] and can play key roles in cancer axes [5]. In addition, circRNAs are supposed to be involved in a variety of molecular mechanisms, such as interactions with RNA-binding proteins [13].

Library enrichment protocols using ribosome depletion and RNA deep sequencing allowed discovering of more than 10,000 human circRNAs having differential developmental stage- and tissue-specific expression. The abundance, pervasiveness, evolutionary conservation and stability of circRNAs, along with emerging evidence of putative circRNA functions, prompted the interests of the scientific community, which generated an array of tools for circRNA analysis [5]. CircRNA detection from RNA-seq data is based on the identification of sequence reads spanning the back-splice junctions generated in circRNAs biogenesis. Back-splice reads map to the genome in chiastic order, i.e., two segments of a single read align separately in reverse order. Thus, circRNA detection from RNA-seq reads needs appropriate methods for non-collinear read alignment and analysis. Recently, several computational methods for the detection of back-splice events from RNA-seq data have been developed, such as find_circ [10], CIRCexplorer [14], circRNA_finder [15], testrealign [16], CIRI [17], KNIFE [18], UROBORUS [19], NCLscan [20], PTESFinder [21], and Acfs [22]. Each type of software uses different strategies for circRNA identification, employing different read aligners, requires variable inputs, as genome and gene annotation, and provides software-related output in term of predicted back-splice junction annotation. Five circRNA prediction tools (circRNA_finder, find_circ, CIRCexplorer, CIRI, and MapSplice [23]) were evaluated for the levels of bona fide and false-positive circRNAs comparing RNase R treated vs. untreated data [24]. This study showed that the most abundant predicted circRNAs are not true positives in all of the cases, that, notably, in general circRNAs identified by one single method lack reliability, and suggested that the combination of at least two methods might be a good practice to increase the robustness of circRNA detection.

In this work, we present CirComPara, an automated bioinformatics pipeline to detect and quantify circRNAs from RNA-seq data using in parallel different methods for back-splice identification. We applied the software to a published RNA-seq dataset, for which circRNA investigation was not previously considered, and provide original results on circRNA expression during monocyte differentiation in relation to the QKI protein function.

## 2. Results

### 2.1. CirComPara Provides circRNA Detection, Quantification, Annotation and Correlation with Gene Expression

We designed and implemented CirComPara (http://github.com/egaffo/CirComPara), a bioinformatics pipeline that allows detecting, quantifying and studying circRNAs from RNA-seq data. CirComPara quantifies linear transcript expression and, in the subsequent phase, it uses in parallel different methods to identify expression of putative circRNAs. The circRNAs are selected according to a combination of the results of circRNA discovery methods, and quantified with normalized estimates. Ultimately, CirComPara annotates the circRNAs in terms of overlapping genes and provides expression correlation measures of circRNA to overlapping genes or linear counterparts. CirComPara yields results in tabular format to ease custom downstream analysis, as well as different default analysis results in HTML pages with several informative display items, such as statistics on circRNAs types, features and expression, and descriptive analyses of samples in terms of circRNA and gene expression profiles, and correlations.

In the next paragraphs, we first describe the software characteristics and then, by a demonstrative application on real data, we show CirComPara’s output.

### 2.2. CirComPara’s Default Workflow and Usage

**Figure 1:**
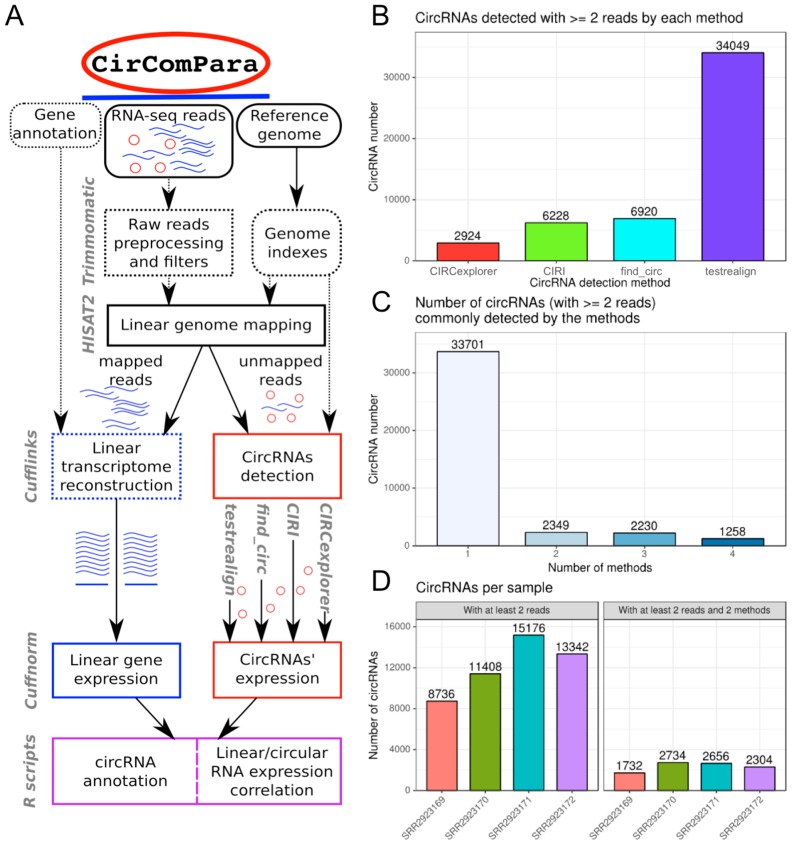
(**A**) CirComPara workflow. Round corner boxes represent inputs; currently used tools are represented by gray labels next to the relative pipeline level; dotted lines represent optional functions; (**B**–**D**) CirComPara summary plots of circular RNAs (circRNAs) expressed; (**B**) absolute number of circRNAs detected by each method and (**C**) commonly detected by two or more methods; (**D**) number of circRNAs expressed per sample, considering the whole set of detected back-splices and the selected subset of circRNAs detected by at least two methods.

A schematic overview of the CirComPara workflow architecture for the analysis of a single sample is represented in Figure 1A. The required input for the pipeline are RNA-seq reads from Illumina sequencing (both single- and paired-end reads supported), the reference genome in multi FASTA format, and the gene/transcript annotation in GTF format (as retrieved from genomic databases like Ensembl). In a typical analysis, the researcher needs to compile a metadata table specifying the sample files and their respective raw read file locations. In addition, the file location of the reference genome and annotation files, which can be stored in a project specification file (vars.py file), together with other non-default parameters chosen.

For each sample, CirComPara first pre-processes the raw reads to retain only high quality fragments that will be used in downstream analysis. In parallel, CirComPara builds the files required by each method, such as genome indexes and specific formats of annotation. Next, a preliminary read alignment to the (linear) reference genome is performed with strict criteria, especially important for paired-end reads: unpaired and discordantly aligned reads are considered unmapped and thus separated from linear aligned reads. The alignments are used to detect and quantify linear transcript and gene expression, which are computed for each sample both as raw counts and normalized values (fragments per kilobase per million mapped reads; FPKM). Reads that fail to be aligned to the reference genome are used as candidates for the detection of back-splice junctions and are given as input to each circRNA detection method. Multiple methods for circRNA detection and quantification can be selected: at the time of this writing, CirComPara uses, as a default setting, four different circRNA detection tools that ground on different strategies (CIRCexplorer, CIRI, find_circ, and testrealign) and unfiltered outputs of each detection tool are saved separately to be available for custom analyses. CircRNAs are distinguished and named in terms of the back-splice genomic positions that identify them. By this approach, predictions from the different methods and samples can be compared and enhanced by defining the set of the “reliable circRNAs”, which, by default, are the circRNAs expressed with at least two back-splice reads and jointly detected by at least two methods.

CircRNAs’ back-splice genomic coordinates are compared against the linear transcripts’ annotation to relate the genes and genomic features that generated circular isoforms. The overlap with gene annotation allows characterizing back-splices according to transcript structure: exonic, intronic, and intergenic circRNAs are the main classes that can be distinguished. Finally, expression levels of the reliable circRNAs (normalized according to reads per million mapped, RPM) are correlated to the overlapping genes’ normalized expression estimates and reported in tabular format.

#### 2.2.1. CirComPara’s Output

CirComPara saves a rich set of results in separate directories. Results of the main analysis steps and objectives are saved in tabular text files, which are useful for data interpretation and post-processing, and offer the user the possibility to examine the complete output. On top, an HTML document reports a detailed summary, with the most relevant results presented in aggregated forms and displayed as tables and figures.

The HTML output file (Supplementary File 1) comprises four different sections. The first and the last sections give technical details, whilst the two central sections, with several subsections each, provide the core information. The first section summarizes the analysis run by listing number and IDs of RNA-seq samples, and the back-splice detection methods used for the combined analysis. The second section is dedicated to statistics and plots reporting the circRNAs detected by at least one program, and circRNAs found only by one method or commonly detected by two or more methods. Next, only bona fide more robust circRNAs detected by at least two methods (reliable circRNAs) are considered for further elaborations, including: circRNA category annotation according to back-splice end positions in relation to known exons or introns of overlapping genes; number of circRNAs expressed by genes; and expression analyses, also in comparison with gene expression profiles. The last section reports names and versions of the software and packages used in the CirComPara analysis run.

#### 2.2.2. Additional Options and Features

CirComPara is a flexible tool that allows output stringency modulation through different setting of parameters. CirComPara by default applies all four circRNA-discovery methods implemented, but different combinations of methods can be specified. In fact, the user can select which circRNA tools to run (at the time of this writing, from one to up to four). For instance, the user may choose only a pair of methods, such as CIRI and find_circ, or find_circ and testrealign; only three methods, such as CIRCexplorer, CIRI, and find_circ; or even only a single method. In addition, the minimum number of back-splice reads and methods jointly predicting a circRNA, which define the reliable circRNA set, can be changed by the user to tune CirComPara’s output. For instance, by setting at least one read and at least one method, CirComPara maximizes its sensitivity, possibly reducing false negative predictions. Conversely, requiring all methods to jointly detect the circRNAs (the most restrictive setting) will possibly reduce the number of false positive predictions.

Moreover, CirComPara can run with alternative workflows. Specifically, raw read preprocessing can be bypassed; already computed genome indexes can be given as input, thus skipping the automatic genome indexes building; and novel genes and transcript isoforms can be inferred from the data when the transcriptome reconstruction option is enabled.

CirComPara optimizes the computational performance by allowing parallel computation at two levels: the former regards a multithreaded run of the tools allowing parallel computing. The latter regards simultaneous execution of CirComPara’s steps that are mutually independent, which maximizes the use of available computational devices when running the program.

### 2.3. CirComPara Predicts Thousands of circRNAs Expressed in Monocytes and Macrophages of a QKI Haploinsufficient Patient and Her Sibling

In the following, we present the pipeline output obtained from a run in default mode on a published dataset, as a demonstrative analysis. We analyzed the RNA-seq data produced by de Bruin et al. [25], which focused on the role of Quaking in linear pre-mRNA splicing in the context of monocytes to macrophage differentiation. As the original study did not interrogate circRNAs, our results illustrate CirComPara discovery power and value.

The dataset comprised four samples of primary monocytes from peripheral blood and experimentally induced differentiated macrophages from a QKI haploinsufficient patient (with 50% QKI mRNA and protein expression due to an unbalanced reciprocal translocation hitting the QKI gene) and her sibling (as control QKI wild-type).

Considering the four samples together, as many as 39,538 non-redundant back-splices were detected by at least one method, with quite different numbers of events detected by different methods from the same sequencing data (Figure 1B): testrealign detected 34,049 back-splice events (86% of the total detected), CIRCexplorer detected 2924 events (7%), whereas CIRI and find_circ detected similar intermediate numbers of back-splices, respectively 6228 (16%) and 6920 (18%). In the *QKI* dataset, 5759 (14.6%) back-splices were detected by a least two methods (Figure 1C) and 85% (33,779) by one method only. Hansen et al. [24] showed that, in RNase R treated libraries, the circRNAs detected by at least two methods are enriched with respect to circRNAs detected by only one method. Although circRNAs detected by only one method could represent true positives, in this sample analysis, we considered circRNAs detected independently by two or more methods, according to CirComPara’s default settings, to reduce false positive calls. Thus, we obtained a set of fairly reliable 5759 circRNAs, which are expressed in monocytes and macrophages (Figure 1D), with apparent quantitative and qualitative differences between *QKI* haploinsufficient and control samples and between monocytes and macrophages.

**Figure 2:**
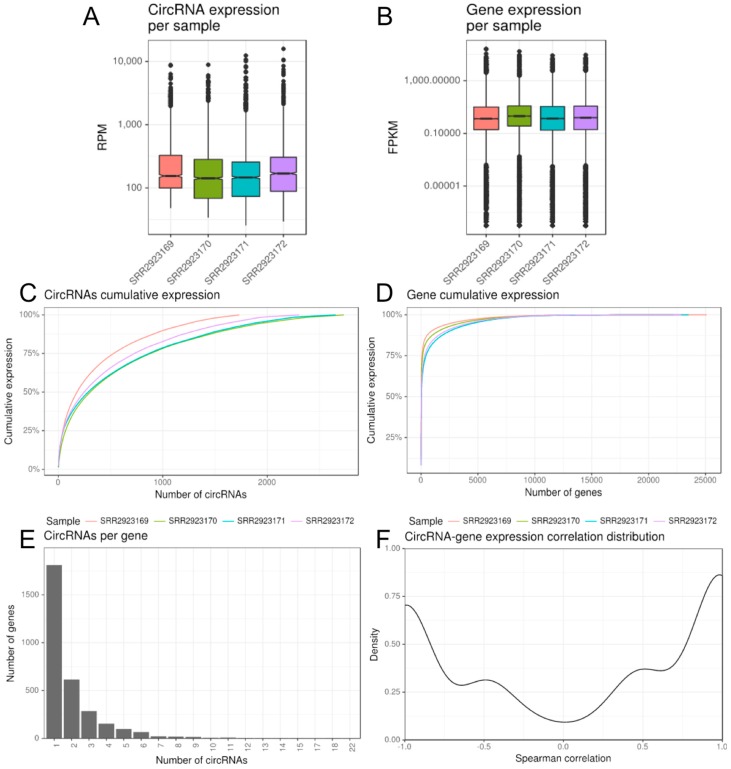
CirComPara summary plots of circRNA and gene expression and integration thereof. (**A**,**B**) twin boxplots of circRNA and gene expression levels per sample; (**C**,**D**) cumulative expression plots of circRNA and gene per sample; (**E**) frequency distribution of number of circRNAs per gene; (**F**) density distribution of pairwise circRNA/gene Spearman correlation values.

CircRNA normalized expression (Figure 2A) values ranged over five degrees of magnitude, reaching over 15,800 RPM in the control macrophages samples for the most abundant circRNA (chr11:33286413–3328751) expressed from *HIPK3* (homeodomain interacting protein kinase 3). CircRNA median expression levels show small differences across the samples, which is also observed in median gene expression, even if with a different pattern (Figure 2A,B). Regarding the cumulative expression, 752 circRNAs on average accounted 75% of sample circRNA expression, whilst only 239 genes on average accounted 75% of sample gene expression (Figure 2C,D).

To evaluate CirComPara’s predictions, we compared predictions from our sample analysis to the 111,665 circRNAs reported by Hansen et al. [24], in which the circRNA expression estimate from fibroblast samples with and without RNase R treatment provided indication on circRNA prediction accuracy. CircRNAs were grouped into: enriched by the treatment (E), probably representing true circular forms; unvaried expression (U); and depleted after treatment (D), probably representing false predictions. The comparison showed that 80% (4619 out of 5759) of the circRNAs selected by CirComPara were also identified by Hansen et al. [24], with 91.6% (4233) of these being enriched, 4.3% (200) being unvaried, and 4.0% (186) being depleted. Table 1 reports the 30 most expressed circRNAs according to de Bruin et al. [25] data. All of these were also detected by Hansen et al. [24], and classified as enriched (26), or unvaried (only four). Furthermore, 14 circRNAs of Table 1 were reported in circBase [26] as experimentally validated in previous studies, including the circRNA from *HIPK3* that was recently functionally characterized as a multiple miRNA sponge [27]. In addition, three of the most expressed circRNAs of Table 1 were reported and characterized in other independent studies [28,29,30].

The 5759 reliably expressed circRNAs resulted associated to 3123 annotated genes, with two circRNAs per gene in average and 42% of circRNAs overlapping genes associated with 2 to up to 22 different circRNAs each (Figure 2E). Indeed, the *PICALM* gene (coding also the Phosphatidylinositol Binding Clathrin Assembly Protein) expresses 22 different circular isoforms, five of them being more abundant and expressed in all four samples. The large majority (5620, 97.6%) of reliable circRNAs resulted exonic and only few were “intergenic” or intronic (with back-splice ends falling outside annotated genes or annotated exons, respectively).

Expression correlation of circRNAs with genes presented a slight tendency toward positive values (Figure 2F): the average and the median values of the 2149 computable pairwise Spearman correlations were 0.07 and 0.40, respectively, and 835 (39%) and 676 (32%) circRNA/gene pairs had a positive correlation over 0.5 or a negative correlation lower than −0.5, whereas 638 (30%) had an (absolute) weaker correlation.

**Figure 3:**
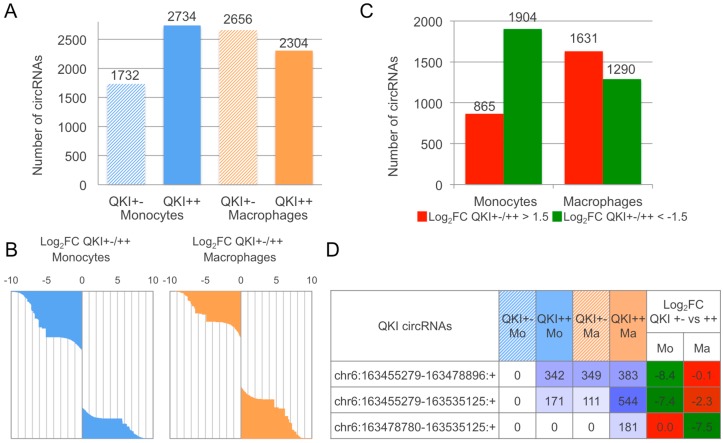
circRNAome expression varies in relation to Quaking (QKI) haploinsufficiency during monocyte to macrophage induced differentiation. (**A**) number of circRNAs expressed per sample; (**B**) waterfall plot of log_2_FC in the *QKI* +/− vs. *QKI* +/+ in the two cell types, for all the expressed circRNAs; (**C**) number of circRNAs with absolute log_2_FC > 1.5 or < −1.5 when comparing QKI haploinsufficient with control cells, separately considering monocytes and macrophages; (**D**) QKI circular isoforms detected from RNA-seq (the table indicates for each circRNA the genomic coordinates of the back-splice ends, the expression level per sample and the intensity of observed log_2_FC).

In addition, we evaluated the number of circRNAs expressed in monocytes and macrophages from the QKI +/− and the control QKI +/+ sibling (Figure 3A). The number of circRNAs expressed is lower (0.63×) in QKI +/− monocytes compared to the control, whereas, in macrophages, the number of circRNAs increases at a lower extent (1.15×) in the same comparison. In accordance with the original study of linear transcripts, and considering that no replicates were available, we calculated the log_2_FC of circRNA expression, considering for each cell type *QKI*+/− versus control values. The expression variation, represented in Figure 3B as waterfall plots of log_2_FC for all expressed circRNAs, is toward downregulation in QKI haploinsufficient monocytes and slightly toward downregulation in macrophages. The number of circRNAs showing a Log_2_FC over 1.5 or lower than −1.5 (Figure 3C) is also informative and is in accordance with the above observations. Indeed, considering only those circRNAs with absolute log_2_FC > 1.5, we identified 865 and 1904 circRNAs up and downregulated when comparing QKI haploinsufficient monocytes. The same comparison regarding macrophages detected 1631 and 1290 up and downregulated circRNAs.

Finally, as an example of analysis that can be performed from CirComPara’s output data, we report in Figure 3D the coordinates and expression values of three circular isoforms expressed from the *QKI* gene showing different abundance in normal monocytes and macrophages that appeared to be affected by the *QKI* haploinsufficiency. All three of these circRNAs resulted as being enriched by RNase R according to the data of Hansen et al. [24] and two of them (6:163455279–163535125; 6:163478780–163535125) were previously detected by Rybak-Wolf et al. [6].

## 3. Discussion

Research advancements on the analysis of RNA-seq data is generating new protocols [31] and improvements of software tools, including methods for circRNA detection [22]. CirComPara was designed and implemented with independent modules interfacing each other in hierarchical scripts. This gives CirComPara great flexibility for incorporating new features, such as additional methods; for updating the used software; and for upgrading to improved performing tools implemented in the pipeline steps.

CirComPara implements practices that, in our opinion, are the current best for the analysis of RNA-seq data and for circRNA discovery. The initial raw read pre-processing step for trimming and filtering low quality reads is highly recommended, although optional, since it was demonstrated to improve the alignment rate, decrease the timings of subsequent processing, and eventually improve the quality of results [32].

The strategy of mapping RNA-seq reads to the linear genome before circRNA identification, also used by other methods [14,33], provides the possibility to characterize gene or transcript expression, reduces computational load for circRNA discovery, and importantly allows for filtering out false positive back-splice findings.

The four circRNA detection methods considered ground on different search strategies used for read alignment to the reference genome, including Burrows-Wheeler [34,35,36] and suffix arrays [16,37] based methods. The combined use of different methods is expected to increase the sensitivity of the overall procedure to the price of an increased number of false positive predictions. The huge number of circRNAs reported by testrealign and the difference with the other methods (nearly five times more circRNAs) is probably due to the fact that, differently from the other methods, testrealign does not perform any alignment post-processing specific for circRNA identification. Such behavior has also been reported recently by [22]. However, by combining the methods’ results and selecting the circRNAs commonly identified by more tools, our method is expected to reduce the number of loose predictions.

CirComPara includes a branch for de novo transcriptome reconstruction and quantification from RNA-seq data in parallel to circRNA detection. Importantly, this feature extends CirComPara application also to species without gene annotation or with scarcely annotated transcriptomes.

From a technical point of view, CirComPara relieves the user from the burden of many preparatory steps that are required by the different tools in the pipeline, such as the read aligners’ genome index building and gene annotation formatting. Moreover, CirComPara provides plentiful access to each method’s optional parameters. In addition to the above mentioned alternative analysis workflows, advanced users can set and combine the parameters to optimize performance and adapt the analysis to specific data. Nevertheless, the use of default parameters can bear good quality results, as it was reported in the present demonstrative analysis.

The sample analysis showed the discovery power of the pipeline and the richness and usefulness of obtained results. De Bruin et al. [25] provided evidence that QKI is induced during monocyte differentiation and plays a key role as a dynamic regulator of pre-mRNA splicing and expression profile changes that drive monocyte activation, adhesion and differentiation into macrophages. Our sample analysis identified numerous circRNAs expressed in monocytes and macrophages, with more than one circular isoform expressed by half of genes. Most of the selected set of 5759 circRNAs detected by two or more methods resulted to be exonic. Moreover, comparison with independent data revealed that almost 92% of these circRNAs are detected also in other tissues and enriched by RNase R treatment.

The expression of circRNAs were scattered across five orders of magnitude, with a relatively low number of elements expressed at high level. Although a direct comparison is difficult, it is worth noting that circRNA expression values distribution were less skewed than that of genes: 13% of circRNAs and 0.8% of genes account for three quarters of the total expression. Notably, almost all 30 most expressed circRNAs reported in Table 1 resulted either being enriched in RNase R treated libraries, and/or experimentally validated by independent studies, such as the top ranking circRNA we detected, which derives from the *HIPK3* gene. A recent study [27] reported four circular isoforms of HIPK3, and showed that the predominant circHIPK3 is abundantly expressed in many tissues where it sponges nine different miRNAs, including the tumor suppressor miRNA miR-124. The same study confirmed circularity and stability of circHIPK3 and showed that the silencing of circHIPK3, but not HIPK3 mRNA, significantly inhibits human cell growth. The other two highly expressed circRNAs in Table 1, expressed by *SPECC1* (17:20204333–20205912) and *CDYL* (6:4891713–4892379), were detected by Schneider et al. [28] that identified IMP3-associated circRNAs. Moreover, the circRNA ZNF609 (zinc finger protein 609) was recently shown to regulate AKT3 (AKT serine/threonine kinase 3) expression by sponging miR-150-5p. This evidence supports the validity of predictions selected by CirComPara.

This study provided a first glance on circRNA expression variations in monocyte to macrophage differentiation and in relation to *QKI* haploinsufficiency. Extending the results of de Bruin et al. [25], we showed indeed that also circRNA expression is affected by *QKI*, with a complex pattern during monocyte to macrophage differentiation, as observed for linear isoforms. *QKI* haploinsufficiency in monocytes seems to reduce the total number of circRNAs expressed respectively to the control and produce a larger number of downregulated circRNAs than of upregulated circRNAs. This result is in accordance with a previous study that showed that QKI promotes circularization [8] and also with de Bruin et al. [25] results on linear RNAs. Indeed, *QKI* haploinsufficiency in *QKI* peripheral blood monocytes was previously associated with significantly lowered expression of targets with QKI response elements (QRE) compared to non-targets (no QRE).

We showed that in macrophages the number and the average expression of circRNAs are moderately increased in relation to *QKI* haploinsufficiency. Also in this case, this is in accordance with the previous observation that macrophages of the haploinsufficient patient showed higher expression of mRNAs containing QREs relative to her sibling. Although preliminary, our results confirm that QKI regulates not only linear splicing but also circular RNA expression during monocyte to macrophage differentiation with a complex pattern. We could hypothesize that QKI regulates the expression of specific circRNAs directly impacting on observed expression patterns. Moreover, QKI regulation of circRNA expression could also be indirect, since QKI could modulate expression of linear RNA isoforms that act or encode *trans*-acting factors involved in circularization. Both direct and indirect interactions deserve further investigation.

## 4. Materials and Methods

### 4.1. CirComPara’s Implementation Details

#### 4.1.1. Automation and Parallelism: Scons

The Scons building tool is the CirComPara’s engine devoted to the automatic execution of the various scripts and tools implementing each step of the pipeline. Scons is a utility software conceived mainly for the compilation of source code in software production, yet its functions can handle tasks beyond standard compiling procedures.

Scons computes the tasks’ execution dependencies, indeed allowing concurrent execution of independent tasks. For instance, CirComPara can simultaneously run the different methods for circRNA detection, but all of them must wait for the termination of the read alignment to the linear genome. In addition, the linear gene/transcript expression quantification is independent from detection of circRNAs, but gene expression to circRNAs’ expression correlation computation depends on both of these steps.

#### 4.1.2. CirComPara’s Software Tools

We used existing methods to implement the analysis steps and developed custom scripts in Python, R and Bash when needed. At the time of this writing, only one method for read preprocessing is supported, Trimmomatic [38], while read statistics are implemented through FASTQC. For the linear genome mapping step, we chose HISAT2 [34] for its speed, accuracy of alignment, low computational requirements, and compatibility with downstream analysis tools, such as Cufflinks [39] and htseq-count [40], that carry out gene and transcript expression quantification. We restrict linear genome alignments by setting HISAT2 parameters to ensure linear and concordant alignments (--no-discordant --no-mixed options). Gene and transcript expression levels are computed with the Cufflinks tool suite, which is also used for the transcriptome reconstruction optional feature. CircRNA detection methods are: CIRCexplorer [14], which uses the STAR [37] aligner; CIRI [17], which uses the BWA-MEM [36] aligner; find_circ [10], which uses the Bowtie2 [35] aligner; and testrealign, from the segemehl [16] aligner. CircRNA annotation was computed by overlapping back splice genomic positions to gene annotation using BEDTools [41]. Analysis report was generated with custom R scripts using the data.table, ggplot2, and knitr R packages. Software versions used for the presented analysis are reported together with references in Table 2.

### 4.2. Quaking Haploinsufficiency Dataset and Analysis Parameters

The sample analysis has been conducted on an RNA-seq dataset (GEO accession GSE74978) recently obtained by de Bruin et al. [25] in a study focused on the role of Quaking in the linear pre-mRNA splicing specifically in the context of monocyte differentiation into macrophages. The dataset comprises four samples (primary monocytes from peripheral blood and differentiated macrophages of a *QKI* haploinsufficient patient and from the control wild-type sibling) for which RNA-seq data (49 million reads per sample in average) have been obtained using Illumina HiSeq 2000 technology, with paired-end design and importantly using a library construction protocol with ribosomal RNA depletion without polyA enrichment, allowing circRNA detection. Samples’ tissue and genotype are reported in Table 3.

Below are listed the CirComPara parameters set for the analysis presented in this work. These parameters were defined in the vars.py file; other non-shown parameters were left with default values: CPUS = “16”, ANNOTATION = “Homo_sapiens.GRCh38.86.gtf”, GENOME_FASTA = “Homo_sapiens.GRCh38.dna.primary_assembly.fa”, PREPROCESSOR = “trimmomatic”, PREPROCESSOR_PARAMS = “MAXINFO:40:0.5 LEADING:20 TRAILING:20 SLIDINGWINDOW:4:30 MINLEN:35 AVGQUAL:30”, CIRCRNA_METHODS = “ciri,circexplorer,findcirc,testrealign”, TOGGLE_TRANSCRIPTOME_RECONSTRUCTION = ‘False’, CUFFNORM_EXTRA_PARAMS = “--output-format cuffdiff”, BWA_PARAMS = “-T 19”.

Adapter sequences used for read preprocessing were from the Trimmomatic file “TruSeq3-PE-2.fa”. The analysis was performed on 64 cores AMD Opteron Processor 6380 with 512 GB of RAM Linux server running 64 bit Ubuntu Precise (12.04.5 LTS).

## 5. Conclusions

RNA-seq data have high discovery potential. The recent possibility of detecting circular RNAs using appropriate methods for back-splice identification extended the set of RNAs that can be studied with RNA-seq assays. CirComPara is a new tool that allows detecting, discovering, and quantifying circRNAs from RNA-seq data using four different methods in parallel for back-splice identification, reporting bona fide predictions from their result comparison. CirComPara also allows for annotating circRNAs in terms of overlapping genes, in order to quantify linear RNAs and gene expression, and to ultimately compare and correlate circRNA with gene and transcript expression levels. Thus, CirComPara is an original method providing substantial improvement for a computationally efficient integrative and comparative study of circular and linear transcriptome from RNA-seq experiments.

## Figures and Tables

**Table 1:** The 30 most expressed circular RNAs (circRNAs) with annotations, estimated expression levels (reads per million mapped reads; RPM), enrichment group in Hansen et al. [24], validation reported in circBase, and references of studies that validated specific circRNAs. E = enriched, U = unvaried, NA = not assayed, VAL = validated.

CircRNA ID	Overlapping Gene Ensembl ID	Overlapping Gene Symbol	CircRNA Category	QKI+− Mo (SRR2923169)	QKI++ Mo (SRR2923170)	QKI+−Ma (SRR2923171)	QKI++ Ma (SRR2923172)	RNase R Enrichment [24]	Validated (CircBase)	Other Studies
11:33286413-33287511:+	ENSG00000110422	HIPK3	exonic	2964	8861	12386	15822	E	VAL	[27]
2:40428473-40430304:−	ENSG00000183023	SLC8A1	exonic	2519	4845	10077	10604	U	NA	
12:108652272-108654410:−	ENSG00000110880	CORO1C	exonic	3090	3625	10701	10471	E	VAL	
17:20204333-20205912:+	ENSG00000128487	SPECC1	exonic	2341	3273	8062	8485	U	NA	[29]
1:7777160-7778169:+	ENSG00000049245	VAMP3	exonic	8613	4941	4105	2832	E|U	NA	
4:143543509-143543972:+	ENSG00000153147	SMARCA5	exonic	4458	6008	4288	4863	E|U	VAL	
14:99458279-99465813:−	ENSG00000183576	SETD3	exonic	5399	2854	5094	6114	E	VAL	
3:196391813-196403019:−	ENSG00000163960| ENSG00000206644| ENSG00000241868	UBXN7|RNU6-1279P| RN7SL434P	exonic|intergenic spanning gene	2848	5594	5553	3853	E	VAL	
8:130152736-130180880:−	ENSG00000153317	ASAP1	exonic	1823	2232	6744	6410	E	NA	
2:201145378-201149835:+	ENSG00000003402	CFLAR	exonic	5294	3675	3775	3644	E	NA	
2:61522611-61533903:−	ENSG00000082898	XPO1	exonic	8801	3638	1503	2329	E	VAL	
1:117402186-117420649:+	ENSG00000198162	MAN1A2	exonic	3498	4779	3812	4127	U	VAL	
8:130358017-130361771:−	ENSG00000153317	ASAP1	exonic	0	250	8392	7447	E	NA	
3:149846011-149921227:+	ENSG00000082996	RNF13	exonic	1044	1278	6891	6829	E|U	VAL	
13:32517857-32527532:−	ENSG00000244754	N4BP2L2	exonic	5770	3191	2788	3627	E	VAL	
18:9182382-9221999:+	ENSG00000265257| ENSG00000101745	RP11-21J18.1|ANKRD12	exonic	5431	3236	3041	2173	U	VAL	
21:15762891-15766141:+	ENSG00000155313	USP25	exonic	3122	2630	2896	4360	E	VAL	
15:64499293-64500166:+	ENSG00000180357	ZNF609	exonic	2770	4645	2109	3334	E	NA	[28]
4:152411303-152412529:−	ENSG00000109670	FBXW7	exonic	3297	4365	2566	2339	E	NA	
4:87195324-87195690:−	ENSG00000145332	KLHL8	exonic	6312	2664	2162	1246	E	NA	
9:110972073-110973558:−	ENSG00000198121	LPAR1	exonic	2039	1168	5094	3715	E	NA	[30]
16:85633914-85634132:+	ENSG00000131149	GSE1	exonic	3340	4629	2089	1859	E	NA	
4:37631385-37638504:−	ENSG00000181826	RELL1	exonic	3469	2260	3336	2653	E	VAL	
6:4891713-4892379:+	ENSG00000153046	CDYL	exonic	1048	1845	3431	4272	E	NA	[29]
5:73074742-73077493:+	ENSG00000157107	FCHO2	exonic	763	1482	4361	3953	E	NA	
5:137985257-137988315:−	ENSG00000031003	FAM13B	exonic	3018	1830	2530	2753	E	NA	
14:73147795-73148094:+	ENSG00000080815	PSEN1	exonic	3881	2625	2055	1408	E	NA	
12:32598497-32611283:+	ENSG00000139132	FGD4	exonic	1579	1639	3884	2764	E|U	NA	
7:158759486-158764853:−	ENSG00000117868	ESYT2	exonic	2734	4661	1026	1042	E	VAL	
8:37765526-37766355:+	ENSG00000147471	PROSC	exonic	1980	2841	2712	1908	E	VAL	

**Table 2:** List of the main software tools included in CirComPara with description of their function, and reference.

Software Tool	Description	Citation/Website	Version
R	custom scripts	http://cran.r-project.org	3.2.5 (2016-04-14)
Python	custom scripts	http://www.python.org	2.7.3
Scons	script execution manager	http://www.scons.org	2.5.0
Trimmomatic	read preprocessing	[38]	0.36
FASTQC	read statistics	http://www.bioinformatics.babraham.ac.uk/projects/fastqc/	0.11.5
HISAT2	linear genome mapping	[34]	2.0.4
CIRCexplorer	circRNAs detection	[14]	1.1.10
STAR	reads alignment by CIRCexplorer	[37]	2.5.2a
CIRI	circRNAs detection	[17]	2.0.2
BWA	reads alignment by CIRI	[36]	0.7.15-r1140
find_circ	circRNAs detection	[10]	1.2
Bowtie2	reads alignment by find_circ	[35]	2.2.9
testrealign	circRNAs detection	[16]	0.1
Segemehl	reads alignment by testrealign	[16]	0.2.0-418
Cufflinks	gene/transcript expression quantification and transcriptome reconstruction	[39]	2.2.1
BEDtools	genome coordinates comparison	[41]	2.26.0
Samtools	handle alignment files; extract unmapped reads	http://www.htslib.org	1.3.1
ggplot2	R library for analysis report	http://ggplot2.org	2.2.0
data.table	R library for analysis report	https://cran.r-project.org/web/packages/data.table/index.html	1.10.0
knitr	R library for analysis report	http://yihui.name/knitr	1.14.0

**Table 3:** Sequence Read Archive (SRA) and Gene Expression Omnibus (GEO) accession numbers, genotype, and cell type of the samples analyzed in the demonstrative analysis.

Sample ID	GEO ID	QKI Status	Cell Type
SRR2923169	GSM1939602	QKI+/−	(CD14+) monocytes from peripheral blood
SRR2923170	GSM1939603	QKI+/+	(CD14+) monocytes from peripheral blood
SRR2923171	GSM1939604	QKI+/−	differentiated CD14+ cells (macrophages)
SRR2923172	GSM1939605	QKI+/+	differentiated CD14+ cells (macrophages)

## Supplementary Materials

The following are available online at www.mdpi.com/2311-553X/3/1/8/s1. File S1 is the result report HTML page obtained from the sample analysis presented in this work.
