# Supplementary material for: CirComPara: A Multi-Method Comparative Bioinformatics Pipeline to Detect and Study circRNAs from RNA-seq Data
Source: Noncoding RNA. 2017 Feb 10;3(1):8. doi: 10.3390/ncrna3010008 (PMC5832002; doi:10.3390/ncrna3010008)
Supplement: Supplementary file 1 [file ncrna-03-00008-s001.html]

CirComPara: detection and analysis of circular RNAs


# CirComPara: detection and analysis of circular RNAs

#### *2016-12-17*

# 1 CirComPara results summary

CirComPara analysis results are presented in this page. Different sections display aggregated data in tables and figures. Raw and integrated results were saved in comma separated (CSV) files.

# 2 Detected circRNA results

Backsplices were detected using 4 programs in parallel: circexplorer, ciri, findcirc, testrealign

The detected circRNAs were saved in the following files:

| sample\_id | file |
| --- | --- |
| SRR2923171 | /home/enrico/QKI/analysis/circrna\_analyze/SRR2923171\_circrna\_norm\_expression.csv |
| SRR2923169 | /home/enrico/QKI/analysis/circrna\_analyze/SRR2923169\_circrna\_norm\_expression.csv |
| SRR2923172 | /home/enrico/QKI/analysis/circrna\_analyze/SRR2923172\_circrna\_norm\_expression.csv |
| SRR2923170 | /home/enrico/QKI/analysis/circrna\_analyze/SRR2923170\_circrna\_norm\_expression.csv |

## 2.1 CircRNAs detected per method

| Method | Tot circRNAs |
| --- | --- |
| testrealign | 34049 |
| findcirc | 6920 |
| ciri | 6228 |
| circexplorer | 2924 |
| Non-redundant | 39538 |

### 2.1.1 CircRNAs shared by methods

|  | circexplorer | ciri | findcirc | testrealign |
| --- | --- | --- | --- | --- |
| circexplorer | 2924 | 2739 | 2550 | 1475 |
| ciri | 2739 | 6228 | 4407 | 2544 |
| findcirc | 2550 | 4407 | 6920 | 2872 |
| testrealign | 1475 | 2544 | 2872 | 34049 |

## 2.2 CircRNAs detected in total

For each method, at least 2 reads were required to consider a circRNAs as detected. Further, circRNAs detected by at least 2 methods are considered more reliable.

| With at least 2 reads | With at least 2 reads and 2 methods |
| --- | --- |
| 39538 | 5759 |

## 2.3 CircRNAs detected by sample

| sample\_id | With at least 2 reads | With at least 2 reads and 2 methods |
| --- | --- | --- |
| SRR2923169 | 8736 | 1732 |
| SRR2923170 | 11408 | 2734 |
| SRR2923171 | 15176 | 2656 |
| SRR2923172 | 13342 | 2304 |

## 2.4 CircRNAs expressed per gene, considering genes expressing circRNAs

The following analysis regards the subset of 5759 detected with at least 2 reads and by at least 2 methods (**reliable circRNAs**).

Number of genes overlapping the 5759 reliable circRNAs: 3123

Number of circRNAs expressed by each gene, with the respective circRNAs IDs, are raported in file /home/enrico/QKI/analysis/circrna\_analyze/circRNAs\_per\_gene.csv (only genes expressing circRNAs are reported).

Distribution of number of circRNAs per gene

|  | Value |
| --- | --- |
| Min. | 1.000 |
| 1st Qu. | 1.000 |
| Median | 1.000 |
| Mean | 2.008 |
| 3rd Qu. | 2.000 |
| Max. | 22.000 |

## 2.5 CircRNA categories detected

Considering reliable circRNAs, the table below indicate the mumber of circRNAs falling in each annotation class according to backsplice start and end positions in relation to annotated exon or introns from overlapping genes.  
NB: circRNA backsplice positions may intersect exon/introns of several genes if multiple genes overlap.

| Category | Tot. circRNAs |
| --- | --- |
| exonic | 5304 |
| exonic|intergenic\_spanning\_gene | 304 |
| intergenic | 118 |
| exonic|intronic | 11 |
| intergenic\_spanning\_gene | 6 |
| intronic|exonic | 5 |
| intronic | 5 |
| intergenic\_spanning\_gene|exonic | 3 |
| intronic|exonic|intergenic\_spanning\_gene | 1 |
| exonic|intronic|intergenic\_spanning\_gene | 1 |
| intronic|intergenic\_spanning\_gene | 1 |

# 3 CircRNA and gene expression

CircRNA expression by sample have been saved in file /home/enrico/QKI/analysis/circrna\_analyze/circRNA\_expression\_per\_sample.csv

Gene expression is reported in file /home/enrico/QKI/analysis/cuffdiff/genes.read\_group\_tracking.

## 3.1 Expressed genes

| sample\_id | Tot. gene expressed |
| --- | --- |
| SRR2923169 | 25100 |
| SRR2923170 | 22164 |
| SRR2923171 | 23517 |
| SRR2923172 | 22875 |
| Non-redundant | 29330 |

## 3.2 CircRNA expression distribution by sample

CircRNA expression (RPM) distribution

|  | SRR2923169 | SRR2923170 | SRR2923171 | SRR2923172 |
| --- | --- | --- | --- | --- |
| Min. | 48.13 | 33.78 | 25.43 | 29.47 |
| 1st Qu. | 99.70 | 68.72 | 73.39 | 88.35 |
| Median | 154.70 | 142.30 | 146.60 | 169.10 |
| Mean | 340.00 | 276.80 | 273.10 | 310.50 |
| 3rd Qu. | 327.50 | 282.10 | 256.60 | 306.50 |
| Max. | 8801.00 | 8861.00 | 12390.00 | 15820.00 |

## 3.3 Gene expression distribution by sample

Gene expression (FPKM) distribution

|  | SRR2923169 | SRR2923170 | SRR2923171 | SRR2923172 |
| --- | --- | --- | --- | --- |
| Min. | 0.000e+00 | 0.000e+00 | 0.000e+00 | 0.000e+00 |
| 1st Qu. | 1.881e-01 | 3.554e-01 | 1.745e-01 | 1.917e-01 |
| Median | 1.282e+00 | 2.024e+00 | 1.327e+00 | 1.532e+00 |
| Mean | 7.551e+01 | 6.485e+01 | 4.137e+01 | 4.810e+01 |
| 3rd Qu. | 9.930e+00 | 1.210e+01 | 1.095e+01 | 1.154e+01 |
| Max. | 2.593e+05 | 1.662e+05 | 8.038e+04 | 8.867e+04 |

## 3.4 CircRNA and gene expression plots

### 3.4.1 Expression distribution

Notes on boxplot decorations:

- notches are used to compare groups; if the notches of two boxes do not overlap, this suggests that the medians are significantly different.
- boxes are drawn with widths proportional to the square-roots of the number of observations in the groups

### 3.4.2 Cumulative expression

## 3.5 Correlation of circRNA and gene expression

| Expressed circRNAs | Exonic or intronic circRNAs |
| --- | --- |
| 5759 | 5635 |

In total, 2149 correlations were computed involving 2079 circRNAs and 1390 genes.

Correlation values were saved in file /home/enrico/QKI/analysis/circrna\_analyze/circRNA\_gene\_expression\_correlation.csv

Correlations distribution

|  | Spearman.correlation |
| --- | --- |
| Min. | -1.00000 |
| 1st Qu. | -1.00000 |
| Median | 0.40000 |
| Mean | 0.07352 |
| 3rd Qu. | 1.00000 |
| Max. | 1.00000 |
| NA’s | 3747.00000 |

Positive correlations distribution

|  | Spearman.correlation |
| --- | --- |
| Min. | 0.0000 |
| 1st Qu. | 0.5000 |
| Median | 1.0000 |
| Mean | 0.7958 |
| 3rd Qu. | 1.0000 |
| Max. | 1.0000 |

Negative correlations distribution

|  | Spearman.correlation |
| --- | --- |
| Min. | -1.000 |
| 1st Qu. | -1.000 |
| Median | -1.000 |
| Mean | -0.806 |
| 3rd Qu. | -0.500 |
| Max. | -0.200 |

# 4 Session info

This page was generated with the following packages version

```
## R version 3.2.5 (2016-04-14)
## Platform: x86_64-pc-linux-gnu (64-bit)
## Running under: Ubuntu precise (12.04.5 LTS)
## 
## locale:
##  [1] LC_CTYPE=en_US.UTF-8       LC_NUMERIC=C              
##  [3] LC_TIME=en_US.UTF-8        LC_COLLATE=en_US.UTF-8    
##  [5] LC_MONETARY=en_US.UTF-8    LC_MESSAGES=en_US.UTF-8   
##  [7] LC_PAPER=en_US.UTF-8       LC_NAME=C                 
##  [9] LC_ADDRESS=C               LC_TELEPHONE=C            
## [11] LC_MEASUREMENT=en_US.UTF-8 LC_IDENTIFICATION=C       
## 
## attached base packages:
## [1] grid      stats     graphics  grDevices utils     datasets  base     
## 
## other attached packages:
## [1] ggplot2_2.2.0     data.table_1.10.0 knitr_1.14       
## 
## loaded via a namespace (and not attached):
##  [1] Rcpp_0.12.7        digest_0.6.10      assertthat_0.1    
##  [4] plyr_1.8.4         gtable_0.2.0       formatR_1.4       
##  [7] magrittr_1.5       scales_0.4.1       evaluate_0.10     
## [10] highr_0.6          stringi_1.1.2      lazyeval_0.2.0    
## [13] rmarkdown_1.1      labeling_0.3       RColorBrewer_1.1-2
## [16] tools_3.2.5        stringr_1.1.0      munsell_0.4.3     
## [19] yaml_2.1.13        colorspace_1.2-7   htmltools_0.3.5   
## [22] methods_3.2.5      tibble_1.2
```
